# Are there differences in the patient-reported medication-related problems among asthma and allergy patients? A community pharmacy survey in Finland

**DOI:** 10.1186/s12889-023-16423-y

**Published:** 2023-08-18

**Authors:** Juha Markus Heikkilä, Paula Bergman, Juha Jantunen, Johanna Salimäki, Marika Pohjanoksa-Mäntylä, Paula Kauppi

**Affiliations:** 1 Division of Pharmacology and Pharmacotherapy, Faculty of Pharmacy, University of Helsinki, Helsinki, Finland; 2 Biostatistics Unit, University of Helsinki, Helsinki University Hospital, Helsinki, Finland; 3 Allergy, Skin and Asthma Federation, Helsinki, Finland; 4 The Association of Finnish Pharmacies, Helsinki, Finland; 5 Pulmonary Department, Heart and Lung Center, Helsinki University Hospital, Helsinki, Finland

**Keywords:** Asthma, Allergy, Medication-related problem, Drug-related problem, Self-management

## Abstract

**Background:**

A medication-related problem is an event involving medication that interferes with desired health outcomes. Those are largely studied among asthma patients, but little is known about medication-related problems among allergy patients. The objective of this study was to determine the most common patient-reported medication-related problems among asthma patients compared to allergy patients during the self-management of diseases. The other objective was to identify how demographic variables and the received treatment information influence reported problems.

**Methods:**

A nationwide survey was conducted in Finnish community pharmacies (*n* = 785) in September 2016. The survey targeted patients buying prescription medicines for asthma or allergy.

**Results:**

Responses were received from 46% of targeted pharmacies from 956 respondents. At least one medication problem was reported by 24% of asthma patients and 12% of allergy patients. The most common problems among asthma patients were having problems taking medicines on time (16%), problems in the administration technique (7%) and in the use of the inhaler (4%). Among allergy patients, 10% reported problems remembering to take medicines on time. Severe asthma and allergy increased the risk for medication-related problems (OR 1.20, 95% CI 1.04–1.40 and OR 1.17, 95% CI 1.0–1.37). A higher age and less education were associated with fewer reported medication-related problems among both patient groups.

**Conclusions:**

Asthma patients reported more medication-related problems than allergy patients. Among both investigated patient groups, remembering to take medicines on time was the most common. Health care professionals should educate younger patients but also older and less educated asthma and allergy patients to recognize and, to solve medication-related problems. In addition, severe asthma patients still need medication counseling.

## Background

A medication-related problem is defined as an event or circumstance involving medication that interferes or has potential to interfere with desired health outcomes [1]. Medication-related problems leading to unfavorable patient outcomes during self-management of the disease are known phenomena among asthma patients [2–11]. However, among allergy patients, this is not widely researched [11–13]. Guided self-management of asthma is well established and recommended under clinical practice guidelines [14–16]. For allergic diseases, there is also increasing evidence showing benefits of guided self-management approaches [17–19].

Counseling, including treatment information, should be actively provided by all health care professionals (HCPs) involved in the patients’ care to enable successful self-management of chronic diseases such as asthma or allergy [14–16]. Patients’ self-management should be supported with individualized written action plans, including guidance on maintenance treatment and on how to act when experiencing an increase in symptoms and medication-related problems [14–16, 19]. Respiratory diseases and allergies are important public health issues in Finland. Therefore respiratory and allergy patients have been particularly focused on by Finnish health care professionals since 1994 when a public health program called the National Asthma Program started [20, 21]. The program was later developed and continued by the National Allergy Program until 2018 [22, 23]. The emphasis of the programs was on the education of physicians, nurses and pharmacists on asthma and allergies to counsel patients on self-management of these diseases to lower the burden of these diseases in Finland [20–24].

Poor medication adherence is a well-known problem among asthma patients [3–5]. The phenomenon is known among both aged [4] and adolescent [5] patients. Based on observations by pharmacists among asthma patients, inappropriate use of medicine by the patients, inappropriate choice of medicine and adverse drug reactions were the most common medication-related problems [3]. Among allergy patients using physician-prescribed antihistamine tablets, it has been found that only a minority of patients were adherent to the guidance given by HCPs [12]. The same phenomena have been recognized among rhinitis patients [13] Additionally, asthma inhalers are often technically suboptimally used, leading to reduced drug deposition in the lungs and poor asthma control [6–11]. Based on pharmacists’ observations, it was identified that one allergy patient out of five had difficulties in using eye drops and understanding the information on package leaflets of the medicinal product [11].

Studies identifying medication-related problems among asthma and allergy patients are commonly based on register data [2, 12] or on observations by HCPs. [3, 6–11] Only a few studies focusing on medication-related problems are based on patient reports on patients’ perspectives [5]. Additionally, little is known about the association between patients’ sociodemographic characteristics and medication-related problems.

Medication related problems are well known among asthma patients, [2–11] but less is known concerning those among allergy patients [11–13]. Most of the research is based on HCP’s evaluation or based on registers [2, 3, 6–12] hence data from patient perspective is needed. In addition, to our knowledge, there is no previous research comparing asthma and allergy patients from the patient-reported medication problem perspective. Information on medication-related problems among these large patient groups is important from clinical perspective, especially in Finland to follow-up of national level public health programs, [20–24, 26, 27] for HCPs to optimally support patients’ self-management of their diseases.

The objectives of this study were 1) to determine how commonly asthma patients, compared to allergy patients, using prescription medicines for their diseases report preclassified medication-related problems during the self-management of the disease and to identify the most reported problems and 2) to determine how socioeconomic factors and received treatment information, as background variables, may influence the reported medication-related problems.

## Methods

### Design of the survey

The survey was developed in the Skin and Allergy Hospital of Helsinki University using the validated RHINASTMA health-related quality of life questionnaire as a basis [25]. The survey and its earlier versions were also performed earlier by Skin and Allergy Hospital and the Association of Finnish pharmacies in 1998, 2001, 2004 and 2010 [21, 22, 24, 26, 27].

### Respondents and data collection

The present study, as part of a nationwide survey, targeted 5–75 years old asthma and allergy patients acquiring prescription medicines for allergy or asthma from community pharmacies during one week from 5 to 9^th^ September in 2016. The survey was distributed to all pharmacies in Finland (in total to 785 pharmacies) by Association of Finnish Pharmacies. The instructions for the survey were also available before and during the survey in the extranet service accessible for participating pharmacies. A nonbinding guidance was to receive a minimum two responses from smaller pharmacies and 5 responses from bigger pharmacies.

Respondents gave their informed verbal consent to participate in the study to a serving pharmacist while visiting a community pharmacy and filled in the paper questionnaire on site. The parent gave his/her permission and an informed verbal consent for the respondents of 5–17 years and filled in the questionnaire. No identifiable personal data were asked or recorded. Respondents’ following demographic information was collected: year of birth, sex, level of education, occupational status in the labour market, smoking status.

The following background variables related to disease, received treatment information and usage or prescription medicines were collected: the severity of the asthma or allergy (scale from 0 to 10; 0–1 no symptoms, 2–5 mild symptoms, 6–8 moderate symptoms and 9–10 severe symptoms), the source of treatment information (physician, nurse, pharmacist, books, internet, patient organizations), whether the patient had received an individual written treatment plan for asthma or allergy (yes/no), whether the patient used regularly (daily) or irregularly (sporadically or not at all) the asthma or allergy medication during the previous 12 months and whether the patient had received treatment information (the response scale from 0 = no information to 3 = a lot of information)from at least one health care professional (physician, nurse, pharmacist) or from other sources (books, magazine, internet, courses, patient society).

The Rhinasthma questionnaire contained 31 quality of life questions. The symptom score used in the Rhinasthma was from 1 (no worries) to 5 (very bothersome). In addition, this study used the following questions from the survey: whether the patient had 1) problems taking medicines on time (yes/no), 2) problems understanding usage instructions (yes/no), 3) problems in the administration technique (yes/no), 4) problems in the use of the inhaler or device (yes/no), and 5) safety concerns (yes/no).

### Data analysis

An explorative factor analysis was conducted on 31 self-reported Rhinasthma questions to classify the patients into two groups: those with asthmatic symptoms and those with allergic symptoms. Factor analysis was used by Principal Axis extraction method and Varimax with Kaiser Normalization as rotation method. Factor scores obtained from the analysis were used to divide the patients either into current asthma or current allergy patients, based on which of the two factor scores were higher. Questions concerning asthma symptoms and limitations in everyday life (questions 1,2,6,7,8,11, 14–26, 28 and 30) loaded the factor that was labelled as “Asthmatic symptoms”. Questions concerning upper respiratory tract and all other symptoms loaded the factor that was labelled as “Allergic symptoms” [28].

Descriptive statistics were computed showing the frequencies of medication-related problems among asthma and allergy patients. Further descriptive statistics were computed giving percentages of respondents with medication-related problems, compared to those without, classified according to background variables among both patient groups. In addition, descriptive statistics were computed and classified according to the respondents’ different receipt statuses of treatment information. Group comparisons were conducted by using Chi square tests or Fisher’s exact tests and Mann–Whitney U tests depending on variable distributions.

Logistic regression models were conducted to explore associations between background variables (sex, age, level of education, severity of the symptoms, smoking status, receipt status of written action plan and receipt status of treatment information from physician, nurse, and pharmacist) and reported medication-related problems (one or more self-reported medication related problem with asthma or allergy medicines). Moderate-to-severe asthma or allergy (symptom scores 6–10) were compared with mild asthma or allergy (symptoms scores 0–5) as reference. The effects of these variables were first tested univariately with logistic regression models. Variables with statistically significant associations with reported medication-related problems were included in the multivariable logistic regression model. Multivariable models were adjusted for age and sex.

The data were analyzed and computed by using the Statistical Package for Social Sciences (SPSS for Windows Version 24.0, IBM Corporation, Armonk, NY, USA). *P*-values < 0,05 were considered statistically significant.

## Results

### Respondents

**Fig. 1:**
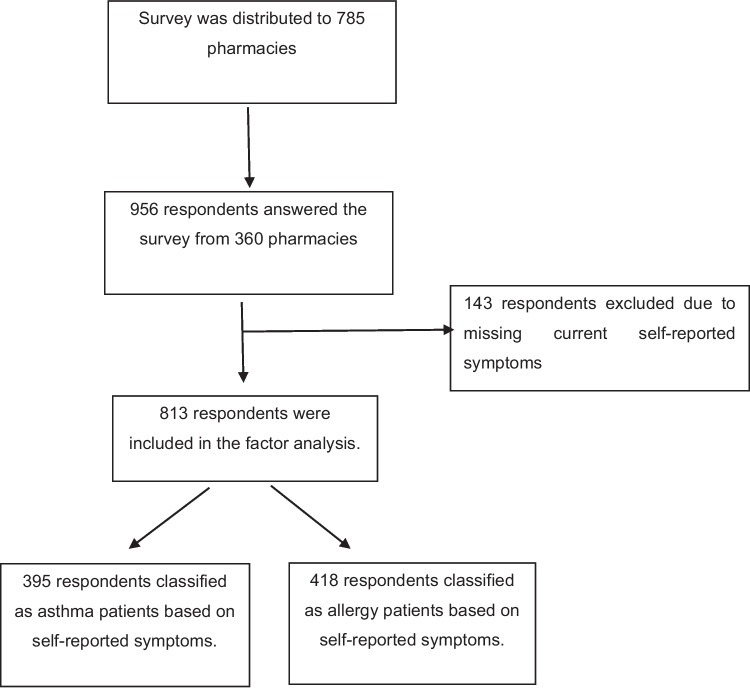
The number pharmacies from which responses were received (*n* = 360) and the number of respondents (*n* = 813) classified as current asthma (*n* = 395) or current allergy patients (*n* = 418) based on their self-reported symptoms

Responses were received from 46% (360) of targeted pharmacies; in total, 956 patients purchased prescription medicines for allergy or asthma (Fig. 1). According to the factor analysis, 395 respondents were classified as current asthma patients and 418 as current allergy patients according to their self-reported symptoms.

The majority of the respondents were female, both among asthma (74%) and allergy patients (75%) (Table 1). The asthma patients were older (*p* < 0,001) and more educated (*p* < 0,001) than allergy patients. There were 70 respondents younger than 18 years of age answered by a parent. There was a statistically significant difference in the working life status between the groups; the majority (57%) of the current allergy patients were in working life, whereas 44% of asthma patients were in working life (*p* < 0,001).

**Table 1:** Characteristics of the current asthma and allergy patients included in the analysis (*n* = 813)

Variable	Current asthma patients, *n* = 395, n (%)	Current allergy patients, *n* = 418, n (%)	*p*-value^a,b^
**Sex**			0.770^a^
Female	292 (74%)	312 (75%)	
Male	103 (26%)	105 (25%)	
Information missing	0 (0.0%)	1 (0%)	
**Age at the time of study (2016)**			< 0.001^b^
5–15 years	16 (4%)	35 (8%)	
16–30 years	47 (12%)	63 (15%)	
31–45 years	54 (14%)	94 (23%)	
46–60 years	122 (31%)	120 (29%)	
61–75 years	156 (39%)	106 (25%)	
**Level of Education**			< 0.001^a^
Primary or secondary level education	115 (29%)	90 (22%)	
Post-secondary or tertiary level education	185 (47%)	167 (40%)	
Bachelor, Master or Doctoral level education	94 (24%)	160 (38%)	
Information missing	1 (0%)	1 (0%)	
**Work life status**			< 0.001^a^
Student	33 (8%)	52 (12%)	
In working life	174 (44%)	237 (57%)	
Outside working life (unemployed, retired or otherwise outside work life)	185 (47%)	120 (29%)	
Information missing	3 (1%)	9 (2%)	
**Smoking status**			0.013^a^
Non-smoker	223 (56%)	280 (67%)	
Current smoker	69 (18%)	54 (13%)	
Ex-smoker	98 (25%)	83 (20%)	
Information missing	5 (1%)	1 (0%)	

a) Chi square test, b) Mann–Whitney U-test. Missing values were not included in the testing procedure

### Medication-related problems

**Fig. 2:**
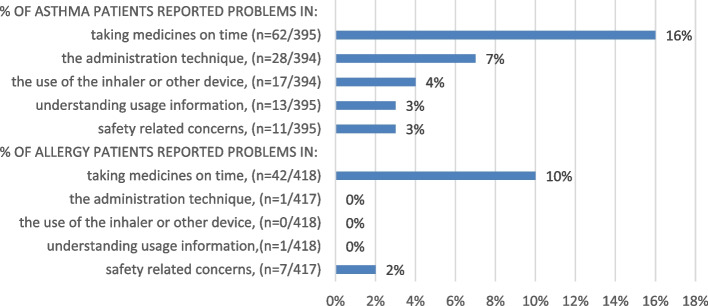
Number and percentages of asthma (*n* = 395) and allergy (*n* = 418) patients answering the survey question on “What of the following issues you have been recognizing to be difficult or hard concerning your medication treatment for asthma or allergies?

Among asthma patients, 24% (*n* = 95) and among allergy patients, 12% (*n* = 50) of respondents reported at least one medication-related problem. The most common problems among asthma patients were problems with taking medicines on time (16%), problems with the administration technique (7%) and problems with the use of the inhaler (4%) (Fig. 2). Among allergy patients, problems remembering to take medicines on time were reported by 10% of respondents.

Younger patients, patients with higher levels of education and patients experiencing more severe symptoms reported medication-related problems significantly (*p* < 0.005) more often than patients with a higher age, lower levels of education and less severe symptoms among both asthma and allergy patients. (Table 2). Whether the respondent had a written action plan or not, usage of medications on a regular or irregular basis or sex did not significantly influence medication-related problems reported by asthma and allergy patients.

**Table 2:** Number and percentages of asthma patients (total *n* = 395) and allergy patients (total *n* = 418) who reported medication-related problems versus asthma and allergy patients who did not report medication-related problems classified according to background variables

ASTHMA PATIENTS, *n* = 395									ALLERGY PATIENTS, *n* = 418								
**Sex**	**female**				**male**			***p*-value**	**Sex**	**female**				**male**			***p*-value**
problems reported, *n* = 96	73 (76%)				23 (24%)			0.56^#^	problems reported, *n* = 50	37 (74%)				13 (26%)			0.90^#^
no problems reported, *n* = 297	217 (73%)				80 (37%)				no problems reported, *n* = 366	274 (75%)				92 (25%)			
**Age**	**median, years**							***p*-value**	**Age**	**median, years**							***p*-value**
problems reported, *n* = 96	49.0 (28.3 – 59.0)							< 0.001^##^	problems reported, *n* = 50	40.5 (26.0 – 49.25)							< 0.001^##^
no problems reported, *n* = 297	58.0 (45.5. – 67.0)								no problems reported, *n* = 367	50.0 (33.0 -62.0)							
**Work life status**	**in work life**				**out of work life**			***p*-value**	**Work life status**	**in work life**				**out of** **work life**			***p*-value**
problems reported, *n* = 95	47 (50%)				48 (50%)			0.25^#^	problems reported, *n* = 50	34 (60%)				16 (40%)			0.09^#^
no problems reported *n* = 295	126 (42%)				169 (58%)				no problems reported, *n* = 365	202 (55%)				163 (45%)			
Education*	**Low**		**Middle**			**High**		***p*-value**	Education*	**Low**		**Middle**			**High**		***p*-value**
problems reported, *n* = 95	23 (24%)		40 (42%)			32 (34%)		0.030^#^	problems reported, *n* = 50	10 (20%)		12 (24%)			28 (56%)		0.015^#^
no problems reported, *n* = 297	92 (31%)		144 (48%)			61 (21%)			no problems reported, *n* = 366	80 (22%)		155 (42%)			131 (36%)		
**Severity of symptoms**	**non-severe**				**severe**			***p*-value**	**Severity of symptoms**	**non-severe**				**severe**			***p*-value**
problems reported, *n* = 96	41 (43%)				55 (57%)			0.002^#^	problems reported, *n* = 49	30 (61%)				19 (39%)			0.03^#^
no problems reported, *n* = 283	172 (61%)				111 (39%)				no problems reported, *n* = 356	271 (76%)				87 (24%)			
**Usage of asthma medication**	**regular**				**irregular**			***p*-value**	**Usage of nasal spray**	**regular**				**irregular**			***p*-value**
problems reported, *n* = 91	82 (90%)				9 (10%)			0.94^#^	problems reported, *n* = 45	16 (36%)				29 (64%)			0.96^#^
no problems reported, *n *= 281	254 (90%)				27 (10%)				no problems reported, *n* = 276	97 (35%)				179 (65%)			
									**Usage of eye drops**	**regular**				**irregular**			***p*-value**
									problems reported, *n* = 28	1 (4%)				27 (96%)			0.22^###^
									no problems reported, *n* = 180	25 (14%)				155 (86%)			
									**Usage of antihistamine tablets**	**regular**				**irregular**			***p*-value**
									problems reported *n* = 50	29 (58%)				21 (42%)			0.54^#^
									no problems reported, *n* = 324	173 (53%)				151 (47%)			
**Written action plan received**	**yes**				**no**			***p*-value**	**Written action plan received, (rhinitis)**	**yes**				**no**			***p*-value**
problems reported, *n* = 96	65 (68%)				31 (32%)			0.64^#^	problems reported, *n* = 49	17 (35%)				32 (65%)			0.35^#^
no problems reported, *n* = 289	203 (70%)				86 (30%)				no problems reported, *n* = 351	99 (28%)				252 (72%)			
									**Written action plan received, (atopic eczema)**	**yes**				**no**			***p*-value**
									problems reported, *n* = 48	8 (17%)				40 (83%)			0.41^#^
									no problems reported, *n* = 324	71 (22%)				253 (78%)			
									**Written action plan received, (food allergy)**	**yes**				**no**			***p*-value**
									problems reported, *n* = 48	7 (15%)				41 (85%)			0.94^#^
									no problems reported, *n* = 313	47 (15%)				266 (85%)			
									**Written action plan received, (risk of anaphylaxis)**	**yes**				**no**			***p*-value**
									problems reported, *n* = 32	3 (9%)				29 (81%)			0.59^#^
									no problems reported, *n* = 186	29 (16%)				157 (84%)			

^*^Education: Low No primary or secondary level education, *Middle* postsecondary or tertiary level education, *High* Bachelor’s, master’s or doctoral level education

^#^Chi square test, ## Mann–Whitney U-test, ### Fisher’s exact test

Both asthma and allergy patients who had received treatment information reported numerically more medication-related problems than respondents who reported having received no treatment information (Table 3). Among asthma patients, those who had received information from HCPs reported significantly more (*p* = 0.015) medication-related problems than those who had received no treatment information from HCPs (Table 3). The same trend was observed among allergy patients, but it was not statistically significant (*p* = 0.10).

**Table 3:** Number and percentages of asthma (total *n* = 395) and allergy patients (total *n* = 418) who reported medication-related problems compared to asthma and allergy patients who did not report medication-related problems classified according to received medication information status and source. # = Chi square test, ## = Fisher’s exact test

**ASTHMA PATIENTS**				**ALLERGY PATIENTS**			
**Medication related problems**	**reported**	**not reported**	***p*-value**	**Medication related problems**	**reported**	**not reported**	***p*-value**
Medication information received from HCP, *n* = 286	85 (30%)	201 (70%)	0.015^#^	Medication information received from HCP, *n* = 309	44 (14%)	265 (86%)	0.10^#^
No medication information from HCP received, *n* = 57	8 (14%)	49 (86%)		No medication information from HCP received, *n* = 61	4 (5%)	57 (95%)	
Medication information received from HCPs, *n* = 286	85 (30%)	201 (70%)	0.10^#^	Medication information received from HCPs, *n* = 309	44 (14%)	265 (86%)	0.065^##^
No medication information received from any source, *n* = 36	6 (17%)	30 (83%)		No medication information received from any source, *n* = 34	1 (3%)	33 (97%)	
Medication information received from any source *n* = 307	87 (28%)	220 (72%)	0.14^#^	Medication information received from any source *n* = 336	47 (14%)	289 (86%)	0.10^##^
No medication information received from any source, *n* = 36	6 (17%)	30 (83%)		No medication information received, *n* = 34	1 (3%)	33 (97%)	
Medication information received from other sources than HCPs = 21	2 (9.5%)	19 (90.5%)	0.7^##^	Medication information received from other sources than HCPs	3 (11%)	24 (89%)	0.31^##^
No medication information received from any source *n* = 36	6 (17%)	30 (83%)		No medication information received from any source *n* = 34	1 (3%)	33 (97%)	

In the multivariable logistic regression analysis, younger age and more severe symptoms were statistically significantly associated with more reported medication problems among the asthma patients and younger age and higher level of education among the allergy patients. (Table 4). Among asthma patients treatment information received from a physician was statistically significantly associated with medication-related problems in the univariable regression model but was not significant in the multivariable model.

**Table 4:** Results of univariable and multivariable logistic regression analysis concerning the background variables of the asthma (*n* = 395) and allergy patients (*n* = 418) having an association with the medication-related problems reported in the pharmacy survey

	ASTHMA PATIETNS, n=395		ALLERGY PATIENTS, n=418	
BACKRGOUND VARIABLE	**Crude Odds Ratio (95% CI)**	**Adjusted Odds Ratio (95% CI)**	**Crude Odds Ratio (95% CI)**	**Adjusted Odds Ratio (95% CI)**
Sex, (ref. Male)	1.17 (0.69; 2.00)	1.00 (0.55; 1.81)	0.96 (0.49; 1.88)	0.94 (0.44; 2.02)
Age, years	0.97 (0.96; 0.98)	0.97 (0.96; 0.99)	0.97 (0.96; 0.99)	0.97 (0.95; 0.99)
Level of Education				
Primary or secondary level education	0.48 (0.26; 0.89)	0.54 (0.27; 1.07)	0.59 (0.27; 1.27)	0.36 (0.14; 0.94)
Post-secondary or tertiary level education (Bachelor, Master or Doctoral level education) (ref.)	0.53 (0.31; 0.92)	0.59 (0.32; 1.08)	0.36 (0.18; 0.74)	0.44 (0.21; 0.93)
Severity of symptoms (0-10) (ref. Mild asthma 0-5)	1.18 (1.06; 1.31)	1.20 (1.06; 1.35)	1.21 (1.04; 1.40)	1.17 (1.00; 1.37)
Smoking, (ref. No)	0.75 (0.40; 1.42)		0.55 (0.19; 1.59)	
Received written action plan, (ref. No)	0.89 (0.54; 1.46)		1.43 (0.67; 3.07)	
Information received from physician, (ref. No)	1.99 (1.24; 3.19)	1.33 (0.78; 2.25)	0.92 (0.46; 1.85)	
Information received from nurse, (ref. No)	0.89 (0.54; 1.46)		0.80 (0.34; 1.88)	
Information received from pharmacist, (ref. No)	1.17 (0.73; 1.88)		1.72 (0.94; 3.16)	
Information received from any source, (ref. No)	2.00 (0.80; 4.92)		5.37 (0.72; 40.18)	

## Discussion

In this study, focusing on patient-reported preclassified medication-related problems among asthma and allergy patients, approximately one out of four (24%) asthma patients and more than one out of ten (12%) allergy patients reported at least one medication-related problem. The most reported medication-related problem among both patient groups was remembering to take medicines on time. Among asthma patients, younger age and more severe symptoms of the disease were associated with reported medication related problems. Among allergy patients lower levels of education and younger age were associated with medication-related problems. Only 7.3% of the respondents were children (younger than 18 years) and thus these results are not specially representative for children.

Non-adherence to asthma medications is a well-known problem [3–5, 29]. No direct conclusion concerning medication adherence can be made based on our findings. However, only few asthma (16%) and even fewer allergy patients (10%) reported problems remembering to always take medicines on time. This may be an underestimate compared to real-life circumstances.

In our study, 7% of asthma patients reported problems with the inhalation technique, and 4% reported difficulties with handling the inhaler. Correct use of the inhaler and a good inhaling technique are crucial in the self-management of asthma. Incorrect use of inhalers is associated with poorer drug deposition in the lungs and a lower probability of asthma control [30]. Compared to earlier research, the findings from our study are more favorable. In earlier research, 11–44% of asthma patients had problems either with the inhalation technique or with the handling technique of the dispenser or both [31]. Our more favorable findings should be critically considered to determine whether patients always recognize handling errors during self-management [10]. Teaching of the inhaler technique and its checking by HCPs as recommended in clinical care guidelines is crucial to enable patients’ self-management [10, 14–16]. In our study, among allergy patients, dispenser and administration technique-related problems related to nasal inhalers or eye drops were not reported. However, in a previous Swedish study, 27% of allergy patients had problems in the use of eye drops when observed by pharmacists [11]. To the best of our knowledge, there are no earlier reports focusing specifically on administration or inhalation technique-related problems with nasal inhalers or handling problems of eye drops among rhinitis patients. In our study, these medication-related problems were not a large burden among allergy patients. However, the findings should be confirmed by objective observations by HCPs.

From earlier studies, it is known that physicians are one of the most common source of treatment information for respiratory patients [28, 32]. In our study, asthma patients with more severe symptoms reported more medication-related problems and the same phenomena as a trend was seen among allergy patients. Most likely, these patients with more severe symptoms meet HCPs, including physicians, more often, which might indicate the observed association between received information from a physician and medication-related problems.

In our study there was no statistically significant association between treatment information received from pharmacist and patient reported medication related problems. However, it is known that pharmacists are a common source for treatment information for patients [28, 32].As prescriptions are valid for two years in Finland and not all asthma and allergy patients might meet a physician regularly, the treatment information and support for self-management by pharmacists might become crucial. It is known that pharmacists focus their counselling for COPD patients, as an example of respiratory patients, on medicinal products and on administration and inhalation techniques [28]. Similarly, community pharmacists are high value for allergy patients as well. Earlier research from Australia found that physician-diagnosed rhinitis patients prefer to buy their allergy medicines without prescriptions [33]. Earlier research shows that with active medication counselling and patient interventions at the community pharmacy level, it is possible to positively impact, for example, adherence, inhalation technique and usage of dispensers among both asthma and allergy patients [34].

According to our study, elderly patients and those with less education reported fewer medication-related problems than younger patients and those with higher education. It is known that the educational attainment of asthma patients correlates with health literacy, which has been shown to have a positive effect on patient outcomes [35]. Our findings are quite the opposite of earlier research, as patients with higher education reported more medication-related problems, which might be due to their better health literacy skills in recognizing problems and reporting those more actively in our survey compared to patients with less education [35]. Among older patients, it is known that medication-related problems are defined as inseparable from the individual’s socioemotional context [36]. There are also signs that medication-related problems are considered to be part of regular medication [36]. Additionally, the importance of asthma self-management skills was not rated as important by elderly patients compared to physicians [37]. From this perspective, there may be underreporting of medication-related problems among older patients in our study.

### Strengths and limitations

To our knowledge, this is the first study comparing medication-related problems among asthma and allergy patients based on patients’ self-reporting. The survey was sent to all Finnish community pharmacies and thus represents a nationwide situation and both rural and urban setting. The response rate was 46% which accords to what is seen in recent pharmacy surveys in Finland [28] but being less than half of the pharmacies it reduces the generalization of the study results. Participation in the study was voluntary for both the customers (patients) and for the pharmacists and no fee was provided for the study. This may also have reduced the participants. Further, the survey was conducted because of practical issues in September instead of May which might have reduced the number of patients, especially allergy patients. Among respondents, females were slightly overrepresented compared to males, and thus the generalization of the results into men should be done cautiously.

Remembering to take medicines on time concentrated on the motivational aspect of the information-motivation behavioral skills model [38]. Therefore, our results in our study cannot be interpreted through two other factors in the model (“Information and knowledge about the need for essential behavior” and “The required behavioral skills to achieve the desired behavior”). Further, we did not assess if the respondents actually did take medicines on time or not or whether they had understood the usage instructions, or if there inhaling and administration techniques were correct or not. Thus, our study results reflect the patients’ perceptions with their asthma and allergy medications more than actual detected problems.

### Implications and future studies

Our study identified that asthma patients reported known medication-related problems, but at a lower rate than researched earlier and more than allergy patients. Allergy patients, instead, reported only a limited number of medication-related problems.

Further studies focusing on medication-related problems during self-management from both the patient perspective and HCP perspective simultaneously are needed. Intervention studies involving HCPs among asthma and allergy patients would be needed to compare whether increased support for self-management of asthma and allergy patients would have an effect, for example, on HCP- and patient-reported clinical outcomes and on health economic outcomes.

## Conclusions

Our study shows that asthma patients self-reported more medication-related problems than allergy patients; a problem to remember to take medicines on time was the most common among both patient groups. Asthma patients also reported problems with administration and inhalation techniques. More severe symptoms of the disease were associated with more medication-related problems among asthma patients.

Higher age and less education were associated with fewer medication-related problems among both asthma and allergy patients. Younger and more educated patients seem still need more specific information and treatment counselling on their medications. In addition, patients with severe asthma need medication counselling.

## Data Availability

The datasets used and/or analysed during the current study are available from the corresponding author on reasonable request.

## Abbreviation

HCP: Health Care Professional
